# PHoral: Effects of carnosine supplementation on quantity/quality of oral salivae in healthy volunteer and in subjects affected by common oral pathologies

**DOI:** 10.1097/MD.0000000000026369

**Published:** 2021-06-25

**Authors:** Michele M. Ciulla, Dino Re, Ettore Gilardoni, Alfonsina D’Amato, Alessandra Altomare, Giovanna Baron, Stefano Carugo, Giancarlo Aldini

**Affiliations:** a Laboratory of Clinical Informatics and Cardiovascular Imaging; b Department of Clinical Sciences and Community Health; c Department of Biomedical, Surgical, and Dental Sciences, Istituto Stomatologico Italiano, Aesthetic Dentistry, School of Dentistry; d Department of Pharmaceutical Sciences “Pietro Pratesi”; e University of Milan, Milan; f Fondazione IRCCS Ca’ Granda Ospedale Maggiore Policlinico di Milano, Italy.

**Keywords:** carnosine, microbiome, oral cavity, periodontal disease, saliva, salivary flow

## Abstract

**Background:**

Diseases of the oral cavity (OC) with an infectious trigger such as caries and periodontal disease are extremely common in the general population and can also have effects at the cardiovascular level. The oral salivary flow, with its buffering capacity, is able to regulate the pH of the OC and, therefore, significantly contribute to the ecological balance of the microenvironment in which the oral microbiome (OM) develops. On the other side, when the quality/quantity of salivary flow is altered it is supposed the disruption of this balance with the potential increase in oral pathogens and triggered diseases. Among the endogenous substances able to exert a significant effect on the salivary flow and its characteristics, carnosine (Car), a dipeptide originally isolated in skeletal muscle, represents, thanks to the known buffering properties, a promising principle.

**Methods:**

We aimed this protocol to evaluate the quantitative/qualitative characteristics of the salivary flow in healthy volunteer subjects (n = 20) and in subjects suffering from common OC pathologies (n = 40), before and after 7 days of supplementation with SaliflussTM (Metis Healthcare srl, Milan, Italy), a Class I medical device on the market as 400 mg mucoadhesive oral tablets that has Car as the main ingredient.

**Discussion:**

Combining the characteristics of saliva with the OM and comparing them with OC pathologies, we expect to clarify their reciprocal relationship and, using quantitative proteomics techniques, to help clarify the mechanism of action of Car.

## 1 Introduction

The pH of the oral cavity (OC) is a synthetic parameter that underlies a multifactorial process of continuous adjustment including the effect of saliva^[1]^ as a buffer and the contribution of the OC microbiome.^[2]^ In some diseases of the OC an impairment of these adjustment mechanisms is supposed to alter the normal flow of saliva and, consequently, the pH and the oral microbiome (OM)^[3]^ with the expansion of potentially pathogenic strains such as the most cited streptococcus viridans.^[4]^

Carnosine (Car) is an endogenous dipeptide, composed of β-alanine and l-histidine, that was originally discovered in larger amounts in skeletal muscle of some vertebrates, including humans, showing a greater dependence on non-oxidative forms of energy metabolism.^[5]^ This peculiar association with muscular tissue and its pH-buffering properties has led to associate Car with the intracellular acid-base homeostasis of muscles.^[6]^ More recently, the physiological role of Car has been expanded beyond the intracellular buffering properties, supporting a role in sarcoplasmic Ca^2+^ regulation and neutralization of reactive oxygen species (ROS). It is well known that ROS induce the formation of reactive electrophilic carbonyl species by reacting with lipids and sugars which, in turn, react with proteins forming irreversible adducts (AGEs, ALEs, and EAGLEs) and cross-links that may affect the cardiovascular wall matrix that becomes less distensible, especially during the aging process and/or diseases.^[7]^ Thus, it is thought that Car and, indeed, other histidine-containing peptides, may prevent chronic diseases via their anti-inflammatory, anti-oxidative, anti-glycating, anti-ischaemic, and chelating properties.^[8]^ Furthermore, the localization of Car in other tissues such as the brain, olfactory bulb, heart, stomach, pancreas, kidney has suggested further potential uses in preventing, for example, neurodegenerative disorder and cognitive function or the development of type II diabetes.

The OM is a relevant part of the whole human microbiome since it contains several different niches, with distinct microbial communities, colonizing the OC, including not only bacteria but also fungi, viruses, archaea, and protozoa. These communities form a complex ecological system that influences OC and systemic health. Indeed the prevalent oral diseases (OD), namely dental caries and periodontal diseases, are believed to be microbiota-related. Furthermore, several evidences support the theory that many systemic diseases are associated with an altered OM, among these the most frequently associated diseases are metabolic, such as diabetes, cardiovascular and oncological ones.^[9]^ For their prevalence worldwide, among OD, periodontal infections, including gingivitis and chronic periodontitis, are possibly the most prevalent human microbial diseases (HMD).^[10]^

To protect the OC from HMD, in the present project we chose Car as a possible preventive and/or therapeutic principle for its aforementioned multiple biological effects. Thus, we decided to test the safety and efficacy of Salifluss^TM^ (Metis Healthcare s.r.l., Milano, Italy) a 400 mg mucoadhesive oral tablet (13 × 4 mm), that recognize Car as the main ingredient, on healthy volunteer and in subjects affected by common OD.

## 2 Aim of the study

The main objectives of this protocol are to estimate the quantity/quality of oral salivae and OM in healthy volunteers and in subjects affected by common OD, before and after 7 days of treatment with Salifluss^TM^, 1 tablet twice a day. The characteristics of oral saliva (Sal) that will be studied are (a) unstimulated and stimulated (paraffin-activated) salivary flow rates and pH and (b) quantitative proteomics (QP), on selected targets, representing the main proteins/components of OM.

By matching Sal characteristics with OM and comparing them with OD, we do expect to elucidate their mutual relationship; furthermore, using specific bioinformatics tools to analyze the data, before and after treatment with Car, we are looking to see its potentials on preventing/treating OD and, using QP, eventually elucidate the mechanism of action.

The study will take place at the Odontoiatric University Clinic (OUC), Istituto Stomatologico Italiano (ISI) of Milan, Italy, in a prospective, randomized, double-blind, and placebo-controlled fashion.

## 3 Methods

### 3.1 Subjects selection

Among subjects referring to the OUC, we will enroll, on a voluntary basis, n = 40 diseased subjects (Ds), aged 18–40 years having at least 1 of the following OD: (a) dental erosions, (b) caries, and (c) périodontopathies, for example, gingivitis or aggressive/chronic periodontitis.

The criteria for each OD were defined on the basis of commonly accepted grading scores in dentistry. Dental erosions will be counted; for caries, we will adopt the missing and filled dental criteria; for périodontopathies, we will record the probing depth, clinical attachment loss, plaque index, and bleeding on probing using a manual periodontal probe.

A complete medical and dental history will be obtained from all participants; all the volunteers must not present allergies/intolerances to the consumption of Car or take other food supplements or any type of drug treatment (interview). Exclusion criteria will be also smoking, pregnancy/lactation, and the presence of any systemic diseases such as cardiovascular and respiratory, diabetes mellitus, HIV infection, or inflammatory conditions causing non-plaque dependent OD. Patients eligible for the study will return to the OUC after being pre-screened. Before being enrolled in the study, participants will provide written and informed consent for use of their saliva samples and clinical data for scientific research purposes. The study is in accordance with the guidelines of the World Medical Association Declaration of Helsinki.

**Figure 1:**
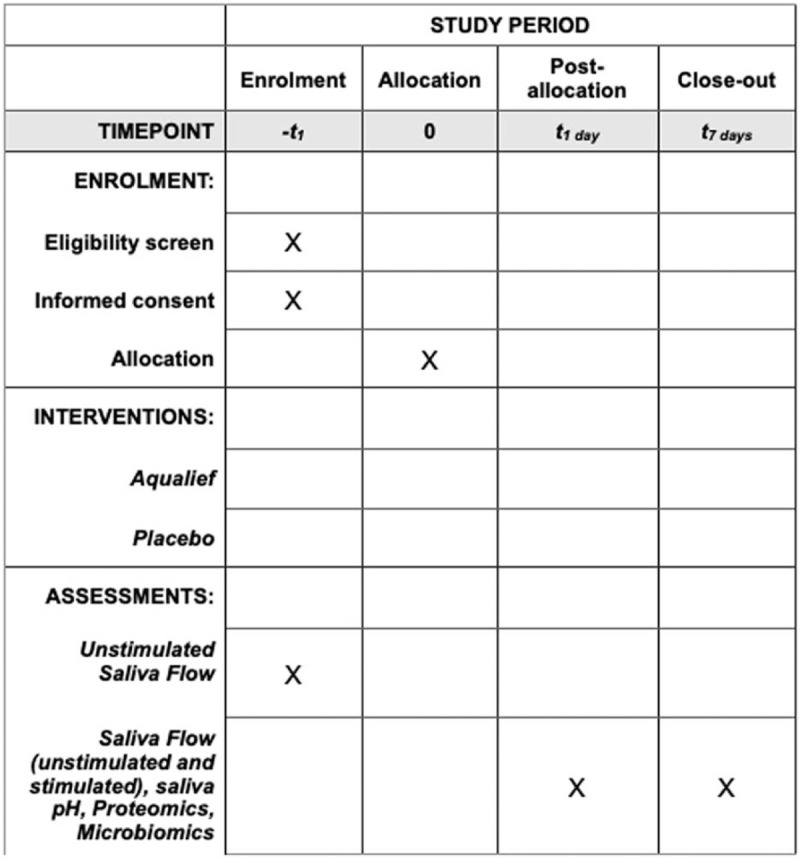
Schedule of enrolment, interventions, and assessments.

**Figure 2:**
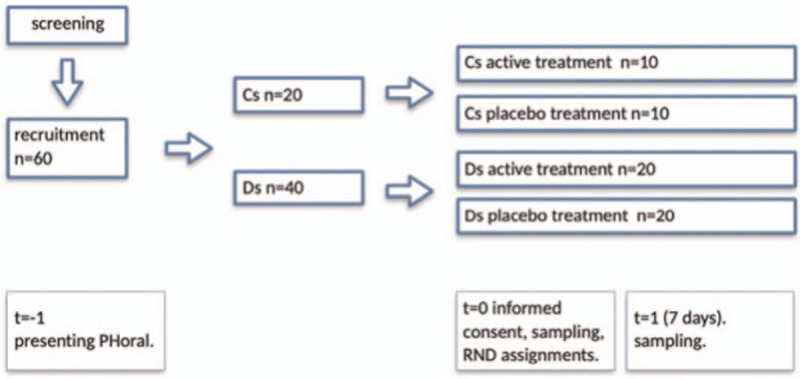
Flow-chart of the protocol.

A control group (Cs) of 20 age-matched subject with periodontal health will be enrolled among the staff of the OUC. The number of subjects identified and considered sufficient for the research is based on a preliminary test conducted on the salivary flow obtained from the staff involved in the project. The schedule of enrolment, interventions, and assessments, and the flow-chart of the protocol are depicted in Figures 1 and 2.

Before the enrolment, patients will read the information and sign the informed consent at the OUC, ISI of Milan, Italy under the supervision of Prof. Dino Re. Participants’ information collected will be confidential.

### 3.2 Sampling

Since the study is focused on saliva samples, subjects with severe hypo-salivation at the first sampling (saliva flow rate at baseline <0.1 mL min^−1^), because of their inability to dissolve the tablet formulation, will be rolled out. Saliva production will be determined by the spit method at baseline (*t* = 0) and after 7 days of treatment (*t* = 1) with Salifluss^TM^ twice a day or placebo.

The participants will be asked to avoid oral hygiene practices including flossing, brushing, and mouth-rinses as well as eating, and drinking for at least 2 hours before collection. Before sampling, the mouth will be rinsed with water for 2 minutes, and the subject will be asked to wait for 10 minutes.

The saliva samples will be obtained in the morning (8.00 am–10.00 am) according to the circadian rhythms in salivary flow.^[11]^ The unstimulated saliva samples will be collected by expectorating into sterile 50 mL tubes every 30 seconds. for 5 minutes as described earlier.^[12]^ The stimulated saliva will be collected in the same way after 2 minutes of chewing a paraffin wax ball.

The samples collected by an operator unaware of clinical data will be weighted, the pH will be measured by a pH-sensitive electrode, then centrifuged at 5000 *g* for 10 min at 4°C; the obtained supernatants will be supplemented with EDTA-free Protease Inhibitor Mixture (Sigma–Aldrich, Dorset, UK) and stored al −80° for the QP analysis. The collected samples will be analyzed at the Department of Pharmaceutical Sciences “Pietro Pratesi,” University of Milan, Milan, Italy (DISFARM) research laboratory.

### 3.3 Administration of Car

Subjects understudy will be instructed to assume Salifluss^TM^ twice a day, in the morning and before bedtime. Salifluss^TM^ is a Class I medical device according to EU Dir. no 93/42/CE (as amended by EU Dir. No. 2007/47/CE), notified to Italian Ministry of Health with ID number 1810197/1810202. At recommended doses, the intake quantities of the substances meet the requirements and limitations set by the Italian Ministry of Health. As previously introduced, Salifluss^TM^ is a 400 mg mucoadhesive oral tablet (13 × 4 mm^2^) with smooth surfaces whose main ingredient is Car. The tablet should be placed in the gingival fornix and, thanks to its mucoadhesiveness, let to dissolve slowly. The dissolution time of the tablet is around 2 hours in normal salivation conditions. Placebo tablets, similar to Salifluss^TM^ ones, will be administered to randomly selected n = 20 subjects, 10/10 from Cs/Ds. An SMS reminder will be sent to all the subject reminding to take the pill.

Patients will be encouraged to report any adverse events and other unintended effects of trial interventions.

Attached the technical and safety data sheets, the certificate of analysis, and the sell-sheet of the extract of Cr.

### 3.4 Proteomics analysis

For proteomics analysis samples stored at −80°C will be thawed and kept on ice before sample preparation. Disulfide bonds of proteins will be reduced using dithiothreitol , and free thiol alkylated with iodoacetamide. Proteins will be digested using trypsin as digesting enzyme. To enhance protein purification, denaturation, and digestion, the above-mentioned passages will be done in S-trap column (PROTIFI, New York, USA). After digestion, peptides will be eluted from the S-trap column, and they will be derivatized with tandem mass tag reagents (TMT kit Thermo Fisher, Milan, Italy). Samples will be further purified, combined for multiplexing analysis, and concentrated. Liquid chromatography–high resolution mass spectrometry analysis will be performed at the Unitech OMICS of the University of Milan, using a Dionex UltiMate3000 nanoflow liquid chromatography (nanoLC) connected in-line with an Orbitrap Fusion mass spectrometer (Thermo Scientific, Milan, Italy).

Data analysis for protein identification and quantification will be carried out with Max Quant software and further filtered with Perseus (Max Plank Institute of Biochemistry, Germany). Networks and pathways analysis will be done with STRING (Swiss Institute of Bioinformatics) and Ingenuity Pathways Analysis (QIAGEN, Hilden, Germany) software.

### 3.5 Statistical analysis

Descriptive statistics of continuous variables understudy will be presented as mean ± SD; categorical variables were expressed as absolute numbers and percentages. The differences in continuous variables will be assessed using the Mann–Whitney *U* test when not normally distributed, and the Student *t* test when normally distributed. The *X*^2^ test was used to test for differences in categorical variables. The relationship between all variables before and after active/placebo treatment will be tested by Spearman correlation coefficients and their 95% confidence intervals.

The primary outcome variable is the treatment-related change in any of the Sal parameter understudy, before and after active/placebo treatment in Cs/Ds subjects by intention to treat criteria, that is, including all patients with adequate sampling before and after treatment (7 days). Differences in treatment-related changes between the groups will be analyzed in the context of covariance analyses (ANCOVA), which included terms for treatment and baseline values as covariates. Treatment effects were assessed by the least-squares mean change from the above ANCOVA model.

If relevant, the intra- and inter-observer variability of sampling, as well as measurements, a repetition of sampling and data analysis will be performed. For intra-observer variability the primary observer will sample/analyze the data on 2 different days; for inter-observer variability the sample/analysis will be performed, independently, by a second observer who was blinded to previous results. Intra- and inter-observer variability will be calculated as the coefficient of variance, which is the standard deviation of the difference between observations divided by their mean and multiplied by 100.

The size of the sample and the power will be calculated expecting a type I error rate of 5% or less. When appropriate, a value of *P* < .05 will be considered significant.

If samples are missing for any cause (eg, not compliance with the trial protocol, no follow-up, others) they will be removed from the analysis.

Data will be analyzed at the end of the study when all samples will be collected. Access to the data in anonymized mode will be provided to the investigators.

A computer statistical package will be used to validate the model and to perform the analysis (SPSS v.20, IBM, Armonk, New York, USA).

## 4 Discussion

From this study, we do expect to elucidate the mutual relationship between Sal characteristics and OM and to compare them with OD; furthermore, using specific bioinformatics tools to analyze the data, before and after treatment with Car, we are looking to see its potentials on preventing/treating OD and, using QP, eventually elucidate the mechanism of Car supplementation.

The study is not yet started, no related publications containing the results of this study have already been published or submitted to any journal; the results of the trial will be shared only by scientific publication.

## Author contributions

All the authors equally contributed to made substantial contributions to the conception, design of the work, the data acquisition, analysis interpretation of data, and revision. All authors have read and approved the manuscript.

**Conceptualization:** Michele M. Ciulla, Dino Re, Ettore Gilardoni, Alfonsina D’Amato, Alessandra Altomare, Giovanna Baron, Stefano Carugo, Aldini Giancarlo.

**Data curation:** Michele M. Ciulla, Dino Re, Ettore Gilardoni, Alfonsina D’Amato, Alessandra Altomare, Giovanna Baron, Aldini Giancarlo.

**Formal analysis:** Michele M. Ciulla, Dino Re, Ettore Gilardoni, Alfonsina D’Amato, Alessandra Altomare, Giovanna Baron, Stefano Carugo, Aldini Giancarlo.

**Funding acquisition:** Aldini Giancarlo.

**Investigation:** Michele M. Ciulla, Dino Re, Ettore Gilardoni, Alfonsina D’Amato, Alessandra Altomare, Giovanna Baron, Stefano Carugo, Aldini Giancarlo.

**Methodology:** Michele M. Ciulla, Dino Re, Ettore Gilardoni, Alfonsina D’Amato, Alessandra Altomare, Giovanna Baron, Stefano Carugo, Aldini Giancarlo.

**Project administration:** Michele M. Ciulla, Dino Re, Ettore Gilardoni, Alfonsina D’Amato, Alessandra Altomare, Giovanna Baron, Stefano Carugo, Aldini Giancarlo.

**Resources:** Michele M. Ciulla, Dino Re, Ettore Gilardoni, Alfonsina D’Amato, Giovanna Baron, Stefano Carugo, Aldini Giancarlo.

**Software:** Michele M. Ciulla, Dino Re, Ettore Gilardoni, Alfonsina D’Amato, Giovanna Baron, Stefano Carugo, Aldini Giancarlo.

**Supervision:** Michele M. Ciulla, Dino Re, Alessandra Altomare.

**Validation:** Michele M. Ciulla, Dino Re, Alfonsina D’Amato, Alessandra Altomare, Giovanna Baron, Stefano Carugo.

**Visualization:** Michele M. Ciulla, Dino Re, Ettore Gilardoni, Alessandra Altomare, Stefano Carugo, Aldini Giancarlo.

**Writing – original draft:** Michele M. Ciulla, Ettore Gilardoni, Giovanna Baron, Aldini Giancarlo.

**Writing – review & editing:** Michele M. Ciulla, Aldini Giancarlo.
